# Thromboinflammatory pathways in breast cancer: clinical and molecular insights into venous thromboembolism risk – a narrative review

**DOI:** 10.1097/MS9.0000000000003644

**Published:** 2025-07-25

**Authors:** Emmanuel Ifeanyi Obeagu

**Affiliations:** Department of Biomedical and Laboratory Science, Africa University, Mutare, Zimbabwe

**Keywords:** breast cancer, coagulation cascade, thromboinflammation, tumor microenvironment, venous thromboembolism

## Abstract

Venous thromboembolism (VTE) remains a significant cause of morbidity and mortality in breast cancer patients, particularly in those with advanced disease or undergoing systemic therapies. While breast cancer is not traditionally classified among the most thrombogenic malignancies, accumulating evidence suggests that tumor biology, treatment modalities, and systemic inflammation can collectively heighten thrombotic risk. This has led to a growing interest in the concept of *thromboinflammation*, wherein the coagulation cascade and inflammatory responses are intricately linked, creating a prothrombotic and tumor-supportive environment. At the molecular level, breast cancer cells and associated stromal elements contribute to a hypercoagulable state through increased expression of tissue factor (TF), release of pro-inflammatory cytokines, and production of TF-bearing extracellular vesicles. Furthermore, components of the innate immune system, particularly neutrophils, promote thrombosis via the formation of neutrophil extracellular traps (NETs). These mechanisms not only facilitate clot formation but also enhance cancer progression, metastasis, and resistance to therapy. Certain aggressive breast cancer subtypes, including triple-negative and HER2-positive tumors, demonstrate heightened thromboinflammatory activity.

## Introduction

Venous thromboembolism (VTE), comprising deep vein thrombosis (DVT) and pulmonary embolism (PE), is a well-recognized complication in cancer patients and a leading cause of mortality second only to cancer progression itself. While malignancies such as pancreatic, gastric, and lung cancers are traditionally associated with higher thrombotic risk, breast cancer – the most frequently diagnosed cancer in women worldwide – is increasingly being acknowledged as a significant contributor to cancer-associated thrombosis. This recognition has been driven by growing clinical evidence of VTE occurrences in breast cancer patients, especially those with advanced-stage disease, aggressive molecular subtypes, or undergoing systemic therapy^[1–3]^. The pathophysiology of breast cancer-associated thrombosis extends far beyond simple vascular stasis or physical compression by tumors. Instead, it involves a complex interplay of hemostatic dysregulation, immune system activation, endothelial dysfunction, and tumor-driven inflammation. This interaction is now framed under the umbrella term “thromboinflammation” – a process where inflammatory and coagulation pathways are synergistically activated, leading to a vicious cycle that supports both thrombosis and cancer progression. In breast cancer, this thromboinflammatory axis is increasingly seen as a critical factor influencing not only thrombotic risk but also clinical outcomes and therapeutic responses^[4,5]^.

In recent years, studies have demonstrated that breast cancer cells can express procoagulant molecules such as tissue factor (TF), which initiates the extrinsic pathway of the coagulation cascade. TF expression is often upregulated by inflammatory cytokines and hypoxic tumor microenvironments. In addition, tumor-derived extracellular vesicles (EVs) that carry TF and other pro-thrombotic molecules have emerged as potent activators of coagulation. These vesicles circulate systemically and can trigger thrombin generation even in distant vascular beds, thereby predisposing patients to VTE^[6,7]^. The immune system also plays a pivotal role in thromboinflammation. In particular, neutrophils are recruited to the tumor site and are primed to release neutrophil extracellular traps (NETs) – networks of chromatin and proteolytic enzymes that ensnare platelets and red blood cells, promoting clot formation. NETs have not only been implicated in thrombosis but also in facilitating tumor cell dissemination and immune evasion. Their presence in the circulation is increasingly being explored as a biomarker of VTE and disease severity in various cancers, including breast cancer^[8,9]^. Another central player in the thromboinflammatory process is the vascular endothelium. In the presence of tumor-derived inflammatory cytokines such as IL-6, TNF-α, and IL-1β, endothelial cells become activated, expressing adhesion molecules and releasing procoagulant factors. This dysfunctional endothelium contributes to a pro-thrombotic environment, characterized by increased platelet adhesion, leukocyte infiltration, and diminished fibrinolysis. In breast cancer patients undergoing chemotherapy or targeted therapy, endothelial injury is further amplified, adding to the thrombotic burden^[10,11]^.

## Aim

The aim of this review is to explore the emerging role of thromboinflammation in breast cancer, with a particular focus on its clinical and molecular implications in VTE risk. This article aims to synthesize current research on the interplay between coagulation, inflammation, and cancer progression, highlighting breast cancer-specific mechanisms of thromboinflammation.

## Review methods

This review was conducted through a comprehensive and systematic approach, with the primary aim of synthesizing the current literature on thromboinflammation in breast cancer, particularly its clinical implications in VTE risk. To ensure the inclusion of relevant and high-quality studies, a multi-step process was followed, incorporating both a broad search of established databases and a meticulous selection of studies that focused on molecular, cellular, and clinical dimensions of thromboinflammation. The review process began with an extensive search of major scientific databases, including PubMed, Scopus, and Web of Science, covering literature published between 2000 and 2025. Keywords such as “breast cancer,” “thromboinflammation,” “venous thromboembolism,” “biomarkers,” and “coagulation” were used in various combinations to capture studies that addressed both the pathophysiological mechanisms and clinical outcomes associated with thrombotic events in breast cancer patients. Articles were selected based on their relevance to the review’s objectives, with preference given to those that provided insights into the molecular underpinnings, biomarkers, and potential therapeutic targets linked to thromboinflammatory pathways. In addition to the database search, manual screening of reference lists from key articles was conducted to identify studies that might have been missed through the initial search. Studies were also selected based on their methodological rigor, ensuring that clinical trials, observational studies, and preclinical models were included to provide a well-rounded perspective. To ensure comprehensiveness, both basic science studies and clinical trials exploring the relationship between breast cancer and VTE were examined. Specific focus was placed on articles that investigated mechanisms such as tissue factor expression, neutrophil extracellular trap (NET) formation, and cytokine signaling, as well as those that explored the role of novel biomarkers and emerging therapeutic interventions.

After the initial selection, articles were screened for inclusion based on predefined criteria. Only studies that addressed the specific aims of the review – namely, those focusing on thromboinflammatory mechanisms in breast cancer and their implications for VTE risk – were included. The full texts of the selected studies were reviewed in detail, with key findings extracted and organized according to the themes of thromboinflammatory mechanisms, biomarkers, clinical implications, and therapeutic strategies. In order to minimize bias and ensure the inclusion of a broad spectrum of evidence, data were extracted and analyzed using a standardized approach. The findings were then synthesized and grouped into thematic sections that align with the objectives of the review. Special attention was paid to emerging trends, such as the growing importance of inflammatory cytokines and extracellular vesicles as biomarkers for VTE risk, as well as the therapeutic potential of targeting thromboinflammatory pathways. The review also adhered to transparent reporting standards, with clear documentation of the methodology, inclusion criteria, and study selection process. Where applicable, quality assessments of the included studies were performed to evaluate their strength and reliability. Limitations of the current literature were also discussed, particularly with regard to the need for more comprehensive clinical studies and the integration of multi-omics approaches in understanding the thromboinflammatory axis in breast cancer.

**HIGHLIGHTS**

- Thromboinflammation bridges cancer and coagulation, where tumor-induced cytokines and tissue factor expression activate clotting cascades, increasing the risk of venous thromboembolism (VTE) in breast cancer patients.
- Platelets and neutrophils actively contribute to cancer-associated thrombosis by forming neutrophil extracellular traps (NETs) and releasing procoagulant microparticles, fueling both metastasis and thrombotic complications.
- Breast cancer subtypes differ in VTE risk, with aggressive phenotypes like triple-negative and HER2-positive showing higher thromboinflammatory activity and prothrombotic profiles, guiding risk stratification.
- Biomarkers such as D-dimer, IL-6, and tissue factor provide molecular clues into thromboinflammatory states, aiding early prediction and monitoring of VTE in breast cancer settings.
- Targeting thromboinflammatory pathways offers dual benefits – reducing clot risk and potentially limiting tumor progression – making anticoagulation therapy a promising adjunct in breast cancer management.

### The thromboinflammatory nexus in breast cancer

The intricate dance between coagulation and inflammation – known as the thromboinflammatory nexus – represents a pivotal element in the progression and complications of breast cancer. Once considered separate physiological processes, inflammation and coagulation are now understood to be deeply intertwined, particularly within the tumor microenvironment. In breast cancer, this relationship is far from coincidental; it is orchestrated by the tumor itself, influencing disease behavior and the risk of venous thromboembolism (VTE)[12]. At the core of this nexus lies tissue factor (TF), a potent initiator of the coagulation cascade. Breast cancer cells, especially in aggressive subtypes like triple-negative and HER2-positive cancers, upregulate TF expression in response to inflammatory signals and hypoxic stress. This TF is not confined to the cell surface – it is also packaged into extracellular vesicles (EVs) that circulate in the bloodstream, acting as mobile procoagulant agents. These vesicles not only initiate clotting but also interact with immune cells and endothelial cells, fueling a cycle of activation and injury[13]. Neutrophils, the first responders of innate immunity, are heavily implicated in this dynamic. Upon activation by tumor-secreted factors or systemic inflammation, neutrophils can release neutrophil extracellular traps (NETs) – web-like structures of DNA and proteases that serve to trap pathogens but, in cancer, entrap platelets and red cells, promoting clot formation. NETs have also been shown to awaken dormant tumor cells, stimulate metastasis, and resist apoptosis, highlighting their dual role in thrombosis and cancer advancement[14]. Adding to this is the role of the vascular endothelium, which becomes a silent accomplice in thromboinflammation. Breast tumors release cytokines like IL-6, IL-1β, and TNF-α, which activate endothelial cells, making them more adhesive and procoagulant. This altered endothelium expresses adhesion molecules such as VCAM-1 and ICAM-1, facilitating leukocyte tethering and contributing to a microenvironment that is primed for thrombosis. Therapies used in breast cancer, including chemotherapy and targeted agents, further damage the endothelium, compounding this risk[15]. The result is a self-sustaining cycle where inflammation begets coagulation, and coagulation fuels further inflammation. This thromboinflammatory loop not only increases the risk of VTE but also supports tumor progression by creating a microenvironment rich in growth factors, matrix degradation enzymes, and immunosuppressive elements. For the patient, this means that a thrombotic event may not just be a complication – it may be a signal of aggressive disease or an early clue to metastasis[16].

### Breast cancer-specific mechanisms of thromboinflammation

While thromboinflammation is a hallmark of many malignancies, breast cancer possesses distinct molecular and clinical attributes that uniquely shape this process. Unlike gastrointestinal or hematologic cancers where overt bleeding or thrombosis is often anticipated, breast cancer presents a subtler but no less significant prothrombotic profile. Its thromboinflammatory mechanisms are finely woven into its molecular subtypes, treatment pathways, and tumor microenvironment dynamics – making them both a challenge to unravel and an opportunity for precise intervention[17]. A defining feature of breast cancer-driven thromboinflammation is the expression of tissue factor (TF) by tumor cells and tumor-infiltrating immune cells. TF acts as the molecular trigger for coagulation, and its expression is enhanced in aggressive subtypes such as triple-negative breast cancer (TNBC) and HER2-positive tumors. Hypoxia, a common condition in rapidly growing breast tumors, further amplifies TF expression, creating a fertile ground for thrombin generation and fibrin formation. What’s particularly unique in breast cancer is the packaging of TF into tumor-derived extracellular vesicles (EVs), which then circulate systemically, instigating clot formation even at distant vascular sites. These EVs act as molecular messengers, delivering both procoagulant and proinflammatory signals[18]. Hormonal influence adds another layer of complexity. Estrogen receptor-positive (ER+) breast cancers are often treated with endocrine therapies like tamoxifen, which, while effective in halting tumor progression, also increase thrombotic risk. Tamoxifen has been shown to alter the balance of clotting factors and promote endothelial activation, subtly nudging the hemostatic system toward hypercoagulability. Moreover, estrogen itself plays a role in regulating coagulation factor expression, platelet function, and inflammatory pathways – mechanisms that can persist even in the post-menopausal context under hormone therapy[19].

Beyond TF, platelets play a central role in breast cancer-specific thromboinflammation. Breast cancer cells release platelet-activating factors, which not only prime platelets for aggregation but also promote the release of platelet-derived microparticles rich in procoagulant and angiogenic mediators. These microparticles contribute to both thrombosis and the establishment of a supportive tumor vasculature. Interestingly, platelets can also “cloak” circulating tumor cells (CTCs), shielding them from immune detection and aiding in metastatic dissemination – a dual role that exemplifies the synergy between clotting and cancer spread[19]. Another critical player is the tumor-associated neutrophil. Breast cancer can manipulate neutrophil function, pushing them toward a pro-tumor phenotype that favors the formation of neutrophil extracellular traps (NETs). These web-like structures not only ensnare circulating tumor cells, facilitating their adhesion to endothelium and eventual extravasation, but also enhance local thrombosis by activating the intrinsic coagulation pathway. The presence of NETs in the circulation of breast cancer patients has been correlated with increased risk of VTE and disease progression[20]. The endothelium in breast cancer is also uniquely responsive to tumor-secreted factors. Vascular endothelial growth factor (VEGF), abundant in many breast tumors, disrupts endothelial integrity and increases vascular permeability. This, combined with systemic cytokines such as IL-6, IL-8, and GM-CSF, leads to endothelial activation – a state characterized by increased expression of adhesion molecules and procoagulant surface proteins. These changes not only promote leukocyte recruitment but also contribute directly to thrombin generation and fibrin deposition[21]. Even the tumor stroma – particularly cancer-associated fibroblasts (CAFs) – contributes to the thromboinflammatory landscape. CAFs secrete matrix metalloproteinases (MMPs) and proinflammatory cytokines that degrade extracellular matrix and facilitate tumor invasion while simultaneously activating platelets and endothelial cells. This matrix remodeling and immune crosstalk form a fertile ground for both cancer progression and microvascular thrombosis[22].

### Molecular insights into venous thromboembolism risk in breast cancer

Venous thromboembolism (VTE), encompassing both deep vein thrombosis (DVT) and pulmonary embolism (PE), is a well-recognized complication in breast cancer patients. However, the molecular mechanisms driving the increased VTE risk in these patients have only recently begun to be understood. Historically, VTE was regarded as a secondary event arising from cancer treatments or advanced disease. Today, research reveals that breast cancer itself, through its complex interactions with the host’s immune and coagulation systems, plays a pivotal role in promoting a prothrombotic state. These interactions, termed thromboinflammation, involve the intricate crosstalk between malignant cells, platelets, coagulation factors, and the immune system, with each contributing to the increased risk of thromboembolic events[23]. A key molecular mechanism contributing to VTE risk in breast cancer is the overexpression of tissue factor (TF), a potent initiator of coagulation. Tumor cells, particularly those with high metastatic potential, can express TF on their surface or release it in microvesicles. This promotes the activation of the extrinsic coagulation pathway, leading to fibrin clot formation. Breast cancer cells, especially those of the aggressive triple-negative subtype, have been shown to exhibit elevated TF expression, making them particularly prone to triggering thrombotic events. Additionally, tissue factor-bearing extracellular vesicles (TF-EVs) are implicated in disseminating procoagulant activity, contributing to thrombosis not only at the primary tumor site but also at distant metastases[24].

Beyond TF, neutrophil extracellular traps (NETs) have emerged as another crucial player in breast cancer-associated thromboinflammation. NETs, web-like structures composed of DNA, histones, and antimicrobial proteins, are released by activated neutrophils in response to inflammatory stimuli. In breast cancer, the presence of tumor-derived signals, such as interleukin-6 (IL-6) and tumor necrosis factor-alpha (TNF-α), can induce neutrophil activation and subsequent NET formation. These NETs are not merely bystanders in the inflammatory response but act as potent facilitators of coagulation, providing a scaffold for thrombus formation. Moreover, NETs have been implicated in enhancing tumor cell adhesion to the endothelium, fostering both thrombotic and metastatic potential in breast cancer[25]. In addition to these cellular mechanisms, inflammatory cytokines play a critical role in shaping the thromboinflammatory landscape. Pro-inflammatory cytokines such as IL-6, IL-8, and TNF-α are commonly elevated in breast cancer and have been shown to directly influence coagulation pathways. These cytokines can upregulate the expression of adhesion molecules on endothelial cells, promoting platelet aggregation and leukocyte adhesion, while simultaneously inducing the expression of procoagulant factors such as TF. In particular, IL-6 has garnered attention for its dual role in promoting both inflammation and coagulation. Elevated IL-6 levels correlate with poor prognosis in breast cancer and are associated with an increased incidence of VTE, further underscoring the inflammatory component of thromboembolic risk[26]. Moreover, the role of the endothelial cell in breast cancer-associated thromboinflammation is pivotal. The endothelium, once considered a passive barrier, is now understood to actively participate in thrombus formation through a process known as endothelial activation. Tumor-secreted factors, including vascular endothelial growth factor (VEGF) and hypoxia-inducible factors (HIFs), can induce endothelial cell dysfunction, increasing vascular permeability and promoting the expression of procoagulant proteins. This disruption of the endothelial layer facilitates the formation of microthrombi, further increasing the risk of VTE in breast cancer patients. Additionally, tumor-induced changes to the blood–brain barrier and other vascular structures may contribute to the heightened susceptibility to thrombosis in advanced stages of disease[27].

### Clinical implications and risk stratification in breast cancer

The interplay between thrombosis and inflammation in breast cancer is not merely a biological curiosity – it carries profound clinical implications. Venous thromboembolism (VTE) is a leading cause of morbidity and mortality in cancer patients, and while traditionally underappreciated in breast cancer, emerging evidence underscores its significance. As our understanding of the thromboinflammatory nexus deepens, so too does the need for robust risk stratification tools that can anticipate VTE events before they manifest clinically, allowing for timely and tailored interventions[28]. Unlike cancers of the pancreas or brain, breast cancer has long been considered a “low-risk” malignancy for VTE. However, such assumptions are increasingly being challenged. With modern treatment modalities – particularly high-dose chemotherapy, targeted therapies, hormonal agents like tamoxifen, and anti-angiogenic drugs – the thrombotic landscape has evolved. Studies now reveal that certain subgroups of breast cancer patients, especially those with aggressive subtypes, advanced-stage disease, or significant comorbidities, face a VTE risk comparable to traditionally high-risk cancers[29]. The clinical implications extend beyond acute thrombotic events. VTE in breast cancer patients is associated with worse overall prognosis, not merely due to the direct consequences of clot formation but also because it may signal a hyperactive tumor biology. Thrombosis can promote metastatic seeding, immune evasion, and even resistance to therapy. Thus, identifying and managing thrombosis risk becomes an integral component of comprehensive cancer care – not an isolated event to be handled in silos[30].

Risk stratification in this context demands more than general prediction models. Tools like the Khorana score, while widely used, were developed with other malignancies in mind and often underestimate thrombotic risk in breast cancer. What is needed are breast cancer-specific risk models that incorporate tumor biology (e.g., subtype, grade), treatment regimen, patient-specific variables (age, BMI, prior VTE), and emerging biomarkers such as tissue factor-positive microparticles, D-dimer levels, C-reactive protein, and NET-related markers. Integrating these factors could yield more personalized VTE risk profiles and guide decisions around prophylactic anticoagulation[31]. From a therapeutic standpoint, stratified care can help balance the benefits of anticoagulation against the risks of bleeding, a particularly crucial consideration in patients undergoing surgery or myelosuppressive therapy. For instance, low molecular weight heparin (LMWH) and direct oral anticoagulants (DOACs) have both shown efficacy in reducing VTE in cancer patients, but their use must be judicious and guided by individual risk. In select high-risk breast cancer patients, prophylactic anticoagulation – when started early – has the potential not only to prevent VTE but possibly to attenuate metastatic spread by disrupting the tumor–coagulation axis[32]. Risk stratification also plays a key role in survivorship care. As breast cancer survival rates improve, clinicians must consider long-term thrombotic risks, particularly in patients receiving extended endocrine therapy. Late-onset VTE, occurring months or even years after diagnosis, can significantly impact quality of life and increase healthcare utilization. Surveillance strategies, guided by initial risk assessment and ongoing clinical indicators, are essential in identifying patients who may benefit from extended monitoring or intervention[33]. Ultimately, the clinical implications of thromboinflammation in breast cancer span the continuum of care – from diagnosis and active treatment to remission and survivorship. By viewing thrombosis not as a complication but as a clinical signal of a more aggressive or inflammatory cancer phenotype, clinicians can refine both oncologic and supportive strategies. This paradigm shift not only improves patient outcomes but aligns with a broader movement toward precision oncology – where treatment is tailored not only to the tumor’s genomic signature but also to its systemic, inflammatory footprint[34].

### Emerging biomarkers and therapeutic targets in breast cancer

The integration of such biomarkers into clinical practice holds promise for transforming the way we stratify, monitor, and treat breast cancer patients at risk of venous thromboembolism (VTE). As the thromboinflammatory link becomes clearer, so does the opportunity to target these pathways therapeutically, moving from generic anticoagulation toward biomarker-guided precision strategies[35]. One of the most promising biomarkers under investigation is tissue factor (TF), especially when embedded in tumor-derived extracellular vesicles (EVs). Elevated levels of TF-bearing EVs in plasma have been associated with both aggressive tumor phenotypes and increased thrombotic events. These vesicles do more than initiate coagulation – they serve as mediators of intercellular communication, transporting oncogenic and proinflammatory signals that promote endothelial dysfunction and platelet activation. Quantifying TF activity within these EVs may serve as both a prognostic indicator and a means to identify patients who could benefit from targeted anticoagulation[36]. Neutrophil extracellular traps (NETs) represent another emerging biomarker and potential therapeutic target. These web-like structures, composed of DNA, histones, and granular enzymes, have been found in the circulation of breast cancer patients and correlate with VTE risk, metastasis, and disease burden. NETs not only promote thrombosis through direct activation of the coagulation cascade but also support tumor progression by facilitating cell adhesion and immune escape. Therapeutics that degrade NETs, such as DNase enzymes, are being explored in preclinical models and may one day serve as adjuncts in high-risk patients[37].

Inflammatory cytokines such as interleukin-6 (IL-6) and interleukin-8 (IL-8) are also gaining recognition for their dual role in promoting tumorigenesis and systemic coagulation. Elevated levels of IL-6 are linked with hypercoagulability, hepatic production of fibrinogen, and poor prognosis in breast cancer. Targeting these cytokines through monoclonal antibodies or small molecule inhibitors may offer a dual benefit – suppressing both tumor-driven inflammation and its thrombotic sequelae[38]. On the endothelial front, soluble P-selectin, von Willebrand factor (vWF), and angiopoietins have been identified as markers of vascular activation and damage, both of which are critical to the thromboinflammatory loop. Monitoring levels of these biomarkers may provide real-time insight into endothelial health and clotting risk, particularly in patients undergoing chemotherapy or anti-angiogenic therapy. Some of these molecules, like P-selectin, are not just passive indicators – they may also be viable targets for therapeutic blockade to prevent platelet-endothelial interactions that precede thrombus formation[39]. In parallel, the use of multi-omics approaches – combining genomics, transcriptomics, proteomics, and metabolomics – is beginning to unravel complex biomarker signatures specific to thromboinflammation in breast cancer. For instance, RNA sequencing has identified upregulated gene sets associated with coagulation and immune signaling in triple-negative breast cancer, while proteomic profiling reveals unique plasma protein patterns linked to clot risk and metastatic potential. These approaches are pushing the frontier of personalized medicine, allowing clinicians to go beyond one-size-fits-all algorithms[40]. From a therapeutic perspective, the next wave of innovation lies in developing agents that modulate thrombosis without impairing hemostasis. Novel anticoagulants that target Factor XIa and tissue factor pathway inhibitors (TFPI) are in early-phase trials, showing potential for cancer-specific application with reduced bleeding risk. Additionally, integrin antagonists, NET inhibitors, and anti-platelet therapies tailored to tumor biology are being explored for their role in disrupting the cancer–coagulation interface[41].

### New insights in breast cancer

Breast cancer, once approached as a localized disease of abnormal cell growth, is now understood to be a complex systemic disorder with far-reaching biological consequences. Modern scientific inquiry has moved beyond the tumor mass to interrogate the intricate crosstalk between malignant cells and the host environment – unveiling new insights that are reshaping how clinicians and researchers view the disease. One of the most transformative revelations lies in the recognition that breast cancer does not evolve in isolation but thrives within a dynamic microenvironment shaped by inflammation, immune responses, and the coagulation system[42]. This evolving paradigm has revealed that the tumor’s influence extends into the bloodstream, activating platelets, coagulation factors, and immune cells in a manner that not only fosters thrombus formation but also promotes metastatic dissemination. It is now evident that breast cancer cells can hijack physiological processes – such as neutrophil extracellular trap (NET) formation, cytokine signaling, and endothelial activation – to create a fertile ground for progression. These processes, once viewed separately, now converge under the unifying framework of thromboinflammation, providing a mechanistic explanation for the observed correlation between cancer progression and thrombotic complications[43]. Concurrently, the molecular subtyping of breast cancer – such as luminal A, luminal B, HER2-enriched, and triple-negative – has offered deeper insight into the biological diversity of the disease. Each subtype exhibits a unique profile not only in terms of growth behavior and therapeutic responsiveness but also in its thromboinflammatory potential. For instance, triple-negative breast cancer, characterized by its aggressive clinical course, also shows heightened expression of procoagulant and inflammatory markers, contributing to a higher VTE risk and poorer prognosis[44].

Innovations in high-throughput technologies, including multi-omics analyses and liquid biopsy platforms, are now enabling researchers to trace the footprints of cancer systemically. Circulating tumor cells, extracellular vesicles, microRNAs, and inflammatory proteins are emerging as real-time indicators of tumor behavior, treatment response, and thrombotic risk. These discoveries are not only transforming diagnostics but are laying the groundwork for individualized treatment approaches that can account for both oncologic and hematologic risks[45]. Importantly, these insights also challenge the long-standing boundaries of cancer care. VTE is no longer viewed merely as a side effect of advanced disease or chemotherapy, but as a biological signature – an early warning sign of a tumor’s systemic aggressiveness. In this context, the oncologist’s role expands to include the management of hemostatic and inflammatory complications, while the hematologist’s understanding of coagulation must now incorporate the nuances of tumor biology[46]. As a result, breast cancer management is entering a new era – one where precision medicine is not just about genetic mutations or receptor status, but about understanding how a tumor interacts with the body at large. From predictive biomarkers to innovative therapeutics targeting the thromboinflammatory axis, the future promises more holistic, risk-adjusted, and patient-centered strategies^[47]^.

## Conclusion

The evolving understanding of thromboinflammatory pathways in breast cancer has opened a critical window into the intersection of cancer biology, inflammation, and hemostasis. No longer seen as a mere complication, venous thromboembolism (VTE) in breast cancer is increasingly recognized as a clinical manifestation of underlying tumor-driven thromboinflammatory activity. This realization marks a paradigm shift – from reactive treatment of thrombotic events to proactive risk stratification and therapeutic targeting of the coagulation–inflammation nexus. Breast cancer-specific mechanisms of thromboinflammation – ranging from tissue factor expression and NET formation to endothelial activation and cytokine dysregulation – highlight the need for precision medicine approaches that consider the tumor’s prothrombotic potential. The emergence of novel biomarkers such as tissue factor-bearing extracellular vesicles, NET-associated components, and inflammatory cytokines promises to refine VTE risk prediction and guide clinical decision-making. Simultaneously, advances in anti-coagulant and anti-inflammatory therapeutics offer hope for tailored interventions that can disrupt the vicious cycle of thrombosis and tumor progression without increasing bleeding risk.

## Data Availability

Not applicable as this a narrative review.

## Research registration unique identifying number (UIN)

Not applicable as this a narrative review.
